# Editorial

**Published:** 1913-11

**Authors:** 


### EDITORIAL

The Business Office of the *Journal-Record* is 23 West Alabama Street.

The Editorial Office is 1014-15 Atlanta National Bank Building.

Address all Business Communications to *Journal-Record of Medicine,* 23 West Alabama Street.

Make remittances payable to *The Journal-Record of Medicine.*

On matters pertaining to the Editorial and Original Communications, address Edgar G. Ballenger, M. D., Atlanta, Ga.

Reprints of Original Articles will be furnished at cost price. Requests for the same should always be made in THE MANUSCRIPT.

We will present, postpaid, on request to each contributor of an original article, twenty (20) marked copies of *The Journal-Record of Medicine* containing such article.

### THANKSGIVING.

It has been duly proclaimed, promulgated, preached and provided for that there should be general Thanksgiving on the 27th when all the people are expected to shape their thoughts to the expression of gratitude for the blessings accrued and to make their minds and hands receptive of greater blessings to come. Thanks and supplications go together and they are not made of empty words. The acceptance of gifts and the return of thanks for them incurs the obligation to be consistent in using them to the best advantage and in correlating to them all the good that the coming year may present.

The past year has been filled with notable results in the study of hygiene and sanitation, and in the advances that have been made in surgery and internal medicine. The physician may be grateful that he can now approach serious conditions with accuracy of knowledge and fulness of judgment where a year ago, he had to deal with empircism and guess work. The steady lowering of the death rate among children shows this as well as the general change for the better in the puerpural state.

The marvellous results of scientific hygiene at the Panama Canal, which is now practically finished, give the Profession every cause for self congratulation and encouragement. There never has been anything like it in all the world, but it gives the surety that no matter what undertaking world politics and economics may suggest, no matter how great the demand upon human thought and endeavor, the physician of today is ready to meet it with almost super care of the lives involved. The laity has recognized this after great sacrifices have been made to demonstrate it and now the medical men are given due power to carry on their work. The medical department has been the one most frequently consulted in that other great engineering feat, the Ashokan water supply to New York and it so governed that a minimun of injury and death resulted.

Clean, wholesome bodies are necessary to good minds, and good minds show their physical origin. The President has shown forceful and direct thought, thoroughly under his control in the management of the Mexican question so that there is the best promise that war with its unavoidable devastations, diseases and deaths will not come to try mens' souls and to prevent a normal growth and development of the nation. A choleric mind or a dull brain such as any disease of the digestive or eliminative organs might induce would, in all probability have had us in war long before this. We would not detract for an instant from the splendid personality of the President. Indeed, we are of the opinion that it is due to his acumen that we are able to say that his confidential companion is Doctor Grayson. No one supposes for an instant that the President is ill and needs attention on that account. He does need to be kept from physical and mental unhygiene conditions and he gives gives this practical testimony in recognition that the medical profession has learned how to supply just such needs.

The coming year will have new and probably complicated things to offer in the way of greater health and comfort to the people. Likely enough there will be many traditions put aside and destroyed. It is our obligation, in receiving favors now, to make ready for the new ones to come, no matter how strange their apparel, nor what old ideas have to be put away in order to welcome them. Such gifts are made conservatively nowadays and one need not fear them for their strangeness, if they have withstood the test of the Profession.

As for ourselves, we feel in closing our sixtieth year, that life is great and glorious and that the Journal's mission of recording and promulgating medical advances and surgical triumphs has brought it into pleasant places. We express our thanks to our contributors and subscribers and according to our text, we are, with a due sense of obligation as to our influence in the medical world, receptive of a continuance and increase of favors from our friends.

R. R. D.

---

No. 1173.

### UNITED STATES CIVIL-SERVICE EXAMINATION.

### CHIEF MINE SURGEON (Male)

December 8, 1913.

The United States Civil Service Commission announces an open competitive examination for chief mine surgeon, for men only. From the register of eligibles resulting from this position in the Bureau of Mines, Pittsburg, Pa., at a salary ranging from $2,400 to $3,600 per annum, and vacancies as they may occur in positions requiring similar qualifications, unless it is found to be in the interest of the service to fill any vacancy by reinstatement, transfer, or promotion.

The duties of the person appointed to fill this position will be to investigate and report upon health conditions in mines and at mining towns, to outline and direct methods of first aid instruction to miners, to attend mine disasters, and to direct and make pathological and physiological studies concerning the effect upon the human system of poisonous gases, death by shock at mines, and similar subjects.

Competitors will not be assembled for examination, but will be rated on the following subjects, which have the relative weights indicated.

| Subjects: | Weights |
|---|---|
| 1. General education and medical training........... | 20 |
| 2. Experience as surgeon and physician to industrial workers .................................... | 30 |
| 3. Postgraduate laboratory and hospital experience on pathological or physical investigations ............ | 30 |
| 4. Publications or thesis on surgery and sanitation.... | 20 |
| Total ................................. | 100 |

Graduation from a medical college of recognized standing and not less than three years' hospital experience among industrial workers are prerequisites for consideration for this position. Special credit will be given under subject 2 for experience in rendering or directing first aid to the injured and in the study of industrial occupational diseases.

Statements as to training, experience, and fitness, are accepted subject to verification.

Applicants must not have reached their fortieth birthday on the date of the examination.

This examination is open to all men who are citizens of the United States and who meet the requirements.

Persons who meet the requirements and desire this examination should at once apply for Form 304, and special form, to the United States Civil Service Commission, Washington, D. C.; the Secretary of the Board of Examiners, Post Office, Boston, Mass., Philadelphia, Pa., Atlanta, Ga., Cincinnati, Ohio, Chicago, Ill., St. Paul, Minn., Seattle, Wash., San Francisco, Cal.; Customhouse, New York, N. Y., New Orleans, La., Honolulu, Hawaii; Old Customhouse, St. Louis, Mo., or to the Chairman of the Porto Rican Civil Service Commission, San Juan, P. R. No application will be accepted unless properly executed and filed, in complete form, with the Commission at Washington prior to the hour of closing business on December 8, 1913. In applying for this examination the exact title as given at the head of this announcement should be used.

Issued October 29, 1913.

